# Proximity Labeling to Identify β-Arrestin1 Binding Partners Downstream of Ligand-Activated G Protein-Coupled Receptors

**DOI:** 10.3390/ijms24043285

**Published:** 2023-02-07

**Authors:** Ya Zhuo, Valeria L. Robleto, Adriano Marchese

**Affiliations:** Department of Biochemistry, Medical College of Wisconsin, Milwaukee, WI 53226, USA

**Keywords:** GPCR, arrestin, CXCR4, proximity labeling, APEX

## Abstract

β-arrestins are multifaceted adaptor proteins that regulate various aspects of G protein-coupled receptor (GPCR) signaling. β-arrestins are recruited to agonist-activated and phosphorylated GPCRs at the plasma membrane, thereby preventing G protein coupling, while also targeting GPCRs for internalization via clathrin-coated pits. In addition, β-arrestins can activate various effector molecules to prosecute their role in GPCR signaling; however, the full extent of their interacting partners remains unknown. To discover potentially novel β-arrestin interacting partners, we used APEX-based proximity labeling coupled with affinity purification and quantitative mass spectrometry. We appended APEX in-frame to the C-terminus of β-arrestin1 (βarr1-APEX), which we show does not impact its ability to support agonist-stimulated internalization of GPCRs. By using coimmunoprecipitation, we show that βarr1-APEX interacts with known interacting proteins. Furthermore, following agonist stimulation βarr1-APEX labeled known βarr1-interacting partners as assessed by streptavidin affinity purification and immunoblotting. Aliquots were prepared in a similar manner and analyzed by tandem mass tag labeling and high-content quantitative mass spectrometry. Several proteins were found to be increased in abundance following GPCR stimulation. Biochemical experiments confirmed two novel proteins that interact with β-arrestin1, which we predict are novel ligand-stimulated βarr1 interacting partners. Our study highlights that βarr1-APEX-based proximity labeling represents a valuable approach to identifying novel players involved in GPCR signaling.

## 1. Introduction

β-arrestin1 and β-arrestin2 (a.k.a. arrestin-2 and arrestin-3, respectively) are multifaceted adaptor proteins that regulate G protein-coupled receptor (GPCR) signaling [1,2,3]. β-arrestins act in concert with GPCR kinases (GRKs) to promote G protein uncoupling and GPCR internalization via clathrin-coated pits [4,5]. β-arrestins also serve as GPCR-regulated signaling scaffolds or adaptors through protein–protein interactions with diverse signaling molecules. This includes Src family tyrosine kinases [6,7,8], MAPKs [9,10,11], E3 ubiquitin ligases and deubiquitinases [12,13], as well as other signaling molecules [14]. Despite the fact that many interacting proteins discovered so far have contributed to a better understanding of β-arrestin function, the full extent remains to be determined.

Several unbiased approaches have been used to discover β-arrestin interacting proteins relative to the activating GPCR. Previous studies have used classical methods such as yeast two-hybrid [10,15] or immunoprecipitation-based mass spectrometry (IP-MS) analysis [16]. However, there are several caveats associated with these approaches. The yeast two-hybrid method may only detect interactions that are not regulated by GPCR activation, whereas the IP-MS approach can detect only strong interactions and is not well suited to detect weak or transient interactions. Recently, proximity-dependent labeling techniques in combination with affinity purification and mass spectrometry have been developed and used to identify novel protein interaction networks in cells [17,18,19]. Proximity labeling requires genetic engineering of a catalytic enzyme to the bait protein, which selectively and covalently attaches a tag on proximal proteins, which then allows for enrichment via affinity purification [20]. Various proximity labeling techniques including tyramide signal amplification [21,22], ascorbate peroxidase (APEX) [17,18,19], and biotin ligase-based identification (BioID) [23,24] have been applied to identify new protein interaction networks with high temporal and spatial resolution. Bio-ID has recently been applied to discover novel interacting proteins of heterotrimeric G proteins [25]. In particular, APEX-based proximity labeling has been extensively studied because of its advantageous rapid reaction kinetics and favorable labeling radius in living cells [20]. This has been applied to discover GPCR-interacting partners in time and space with high resolution [17,19,26,27].

Here, we used APEX2-based proximity labeling coupled with affinity purification and quantitative mass spectrometry to identify β-arrestin1 (βarr1)-interacting proteins promoted by an agonist-activated GPCR. In contrast to previous studies that appended APEX2 in-frame to the C-terminus of GPCRs [17,19,26,27], we appended APEX2 in-frame to βarr1 with the goal of selectively identifying interacting partners at or near the GPCR/βarr1 complex. Appending APEX2 in frame to the C-terminus of βarr1 (βarr1-APEX2) preserved its function as assessed by coimmunoprecipitation with known interacting partners and by its ability to promote agonist-stimulated internalization of the β2 adrenergic receptor (β2AR). We show that βarr1-APEX2 increased labeling of known βarr1-interacting partners following agonist activation as assessed by streptavidin affinity purification and immunoblotting. To identify novel interacting proteins, samples were analyzed by quantitative mass spectrometry following streptavidin affinity purification. We identified several proteins that increased in abundance with high confidence following agonist stimulation compared with the basal condition. Biochemical experiments confirmed two novel β-arrestin-interacting proteins, which we predict are involved in GPCR signaling. We show that βarr1-APEX-based proximity labeling represents a useful approach to identifying novel players in the GPCR signaling network.

## 2. Results

### 2.1. Characterization of β-Arrestin1-APEX2

**Figure 1:**
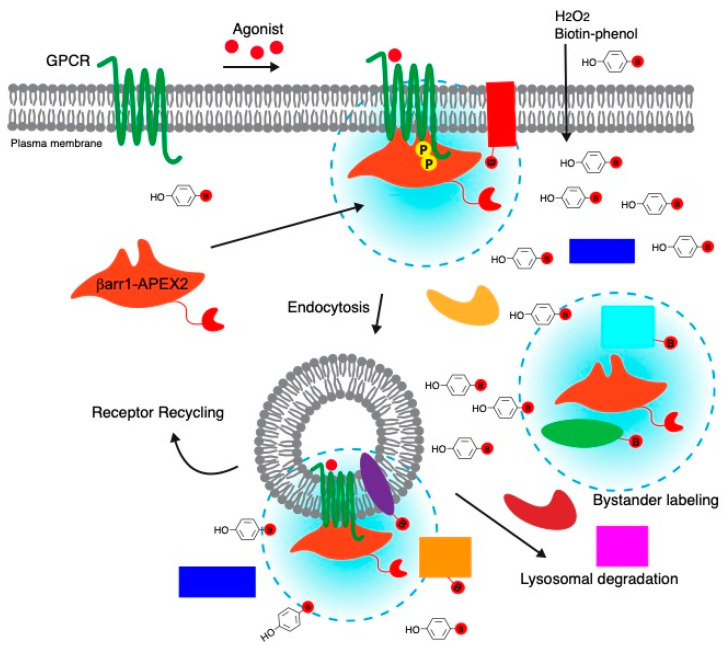
Schematic representation of biotinylation of proximal proteins by β-arrestin1-APEX2 (βarr1-APEX2). βarr1-APEX2 transiently expressed in HeLa cells is mainly diffusely distributed in the cytoplasm under basal conditions. Upon agonist stimulation, βarr1-APEX2 is recruited to ligand-activated and phosphorylated (yellow circle) GPCRs at the plasma membrane and/or endosomes after internalization. After adding biotin-phenol and hydrogen peroxide to the cells, APEX2 rapidly biotinylates proteins proximal to βarr1-APEX2 within a labeling radius of approximately 20 nm. Receptor-bound βarr1-APEX2 is expected to spatially and temporally label proteins at the plasma membrane and/or endosomes. Non-receptor-associated βarr1-APEX2 that remains in the cytosol, similar to APEX2, will label bystander proteins that are located within a 20-nm labeling radius. The labeling radius is indicated by a dotted circle. Proteins within the circles are labeled with biotin (B), and proteins outside of this range are not expected to be labeled. Labeled proteins can be selectively purified by streptavidin-pulldown and analyzed by immunoblotting and/or mass spectrometry.

We used APEX-based proximity labeling coupled with affinity purification and quantitative mass spectrometry to identify the protein interaction network following β-arrestin1 (βarr1) recruitment to an agonist-activated GPCR. The rationale for tagging βarr1 with APEX2 (βarr1-APEX2) is that it would selectively allow us to identify interacting partners after βarr1 has been recruited to the agonist-activated and phosphorylated GPCR (Figure 1). βarr1-APEX in the absence of stimulation (i.e., basal) and APEX2 are expected to be mainly cytosolic and they would serve as spatial controls, which would allow us to distinguish between labeled cytosolic bystander proteins and proteins that were labeled when βarr1-APEX2 was recruited to the GPCR at the plasma membrane following agonist stimulation. In this way, when stimulated with an agonist in a time-dependent manner, we expect to resolve interacting proteins based on their temporal and spatial organization at or near the GPCR/βarr1 complex either at the plasma membrane and/or early endosomes (Figure 1).

**Figure 2:**
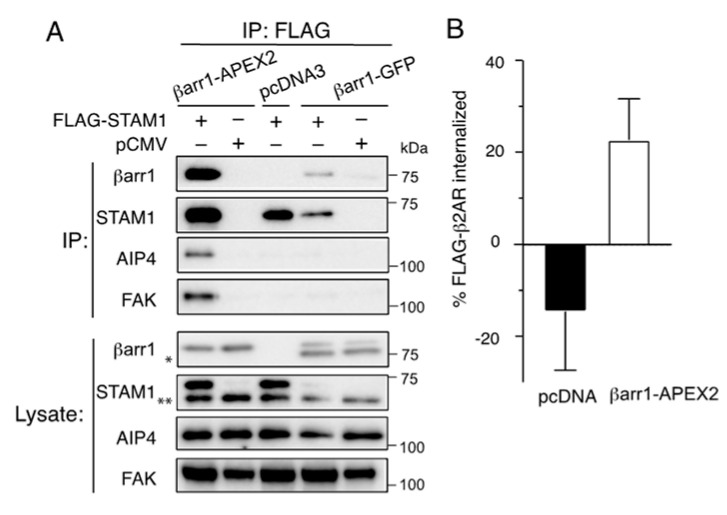
Tagging βarr1 with APEX2 does not interfere with its function. (**A**). Binding of βarr1-APEX2 to known βarr1 binding partners. FLAG immunoprecipitates were prepared from cleared cell lysates of COS-1 cells transiently expressing FLAG-STAM1 or empty vector (pCMV) and βarr1-APEX2, βarr1-GFP or empty vector (pcDNA3). FLAG immunoprecipitates and lysates were analyzed by immunoblotting for the indicated proteins. Shown are representative immunoblots from one of three independent experiments. The single asterisk (*) indicates a degradation product associated with βarr1-GFP. The double asterisks (**) indicate a nonspecific band associated with the antibody against STAM1. FLAG-STAM1 is expressed at low levels when transfected with βarr1-GFP, and not readily visible on the STAM1 immunoblot from lysates on the exposure shown, although present in the immunoprecipitates. (**B**). βarr1-APEX2 rescues internalization of the β2 adrenergic receptor (β2AR) in β-arrestin1- and β-arrestin2-deficient HEK293 cells (βarr1/2 DKO). βarr1/2 DKO HEK293 cells were transiently transfected with FLAG-β2AR and either pcDNA3 or βarr1-APEX2 and were stimulated with either vehicle or 10 µM isoproterenol for 30 min. Cell surface FLAG-β2AR was measured by whole-cell ELISA and receptor internalization was calculated as described in the Materials and Methods Section 4.9. Data represent the mean ± SD from three independent experiments. Data were analyzed by an unpaired t test and the adjusted *p* value = 0.0134.

The ascorbate peroxidase (APEX2) was placed in-frame to the βarr1 C-terminus (βarr1-APEX2), where the placement of other tags has not disrupted β-arrestin function [28]. We first examined whether βarr1-APEX2 interacts with known binding partners by coimmunoprecipitation [29]. For this, we examined the ability of βarr1-APEX2 to interact with STAM1, an adaptor protein whose interaction with βarr1 has been previously shown by multiple approaches [14,29,30,31]. COS-1 cells were transfected with βarr1-APEX2 and FLAG-STAM1 or empty vector (pCMV10). Cleared cell lysates were incubated with an anti-FLAG antibody, and immunoprecipitates were analyzed by immunoblotting. We observed βarr1-APEX2 in FLAG immunoprecipitates, indicating that its ability to bind to STAM1 remained intact (Figure 2A). Furthermore, we detected the presence of other βarr1-interacting partners in the immunoprecipitates that are endogenously expressed in COS1 cells, including AIP4 and FAK, consistent with these proteins also interacting with βarr1-APEX2 (Figure 2A). Furthermore, we confirmed that βarr1-APEX2 is functionally intact by virtue of its ability to rescue agonist-stimulated internalization of the FLAG-tagged β_2_-adrenergic receptor (β_2_AR) transiently expressed in β-arrestin1- and β-arrestin2-deficient HEK293 cells [32,33] (Figure 2B), indicating that βarr1-APEX2 is functionally competent.

### 2.2. Proximity Labeling by βarr1-APEX2 

**Figure 3:**
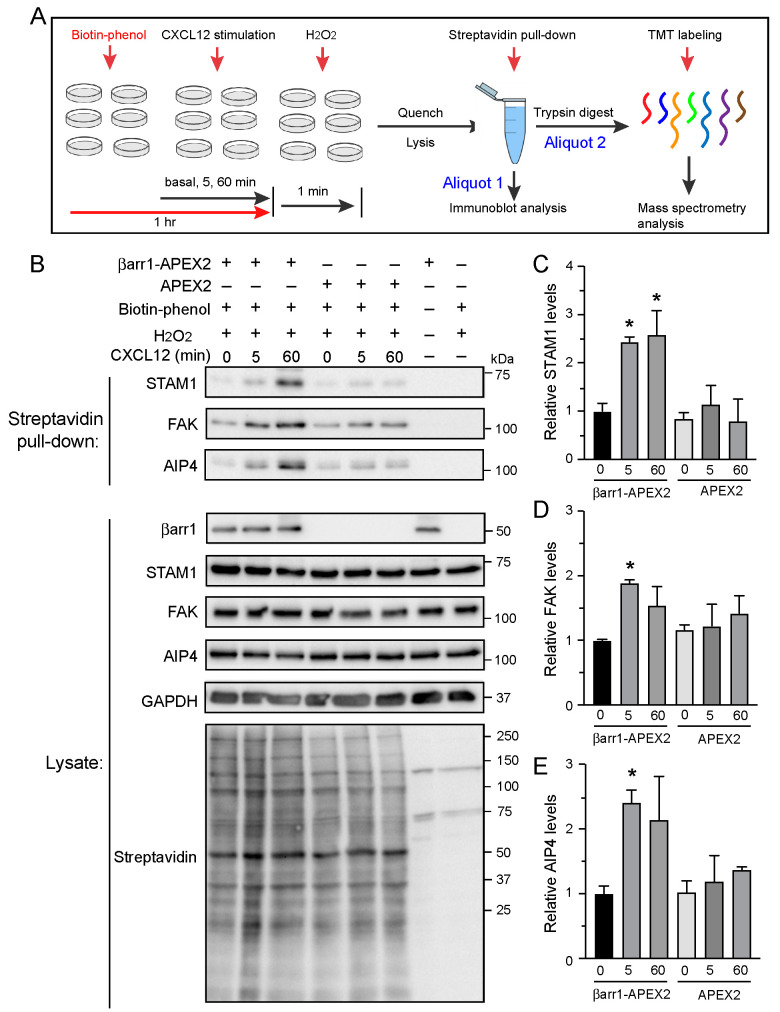
Proximity labeling and experimental workflow for identifying the βarr1 interactome. (**A**) Schematic representation of proximity labeling and experimental workflow. HeLa cells transiently transfected with βarr1-APEX2, APEX2, or empty vector (pcDNA3) were preincubated with biotin-phenol for 1 h. During this time, CXCL12 was added to cells to stimulate endogenous CXCR4 for 5 min or 60 min or not added (basal). Proximity labeling was completed upon hydrogen peroxide treatment for 1 min. After quenching and harvesting, cleared cell lysates were divided into two aliquots. Aliquot 1 was analyzed by streptavidin affinity purification and immunoblotting or while Aliquot 2 was snap-frozen and stored at −80 °C and analyzed by mass spectrometry later. Briefly, aliquot 2 from three biological replicates were processed simultaneously by streptavidin affinity purification, trypsin digestion and tandem mass tag (TMT) labeling for mass spectrometry analysis. (**B**) Immunoblot analysis of proximity-labeled proteins known to interact with βarr1 during the time course of stimulation with CXCL12. Shown are representative immunoblots from one of three independent experiments. (**C**–**E**) STAM1 (**C**) FAK (**D**), and AIP4 (**E**) immunoblots were analyzed by densitometry. Bars represent the mean from three independent experiments normalized to the basal condition (zero). The error bars represent S.D. Data were analyzed by one-way ANOVA followed by Bonferroni’s multiple comparison test. Asterisk (*) indicates adjusted *p* value < 0.05 relative to the basal condition.

The proximity labeling experiments were performed in HeLa cells transiently expressing βarr1-APEX, APEX alone, or empty vector (pcDNA). HeLa cells endogenously express the chemokine receptor CXCR4 [34], which we have previously shown signals via G protein- and β-arrestin-dependent pathways in these cells [29,35]. The experimental workflow for proximity labeling is illustrated in Figure 3A. The experiments were performed under basal and CXCL12-stimulated (50 nM) conditions for 5 min or 60 min. To initiate biotinylation, cells were incubated with biotin-phenol for 1 h followed by the addition of hydrogen peroxide (H_2_O_2_) for 1 min. Reactions were rapidly quenched, cells were extensively washed, and cleared cell lysates were prepared. We divided the cleared cell lysate into two aliquots, whereby one aliquot was snap-frozen and stored at −80˚C to be processed later for mass spectrometry analysis, whereas the other aliquot was processed to verify proximity labeling by streptavidin affinity purification followed by immunoblotting for known βarr1-interacting proteins. Blotting lysates determined the labeling efficiency of βarr1-APEX2 with HRP-conjugated streptavidin. Many proteins were biotinylated by βarr1-APEX2, which did not appear to be influenced by CXCL12 stimulation (Figure 3B). The global pattern of labeling between βarr1-APEX2 and APEX2 appeared similar, suggesting that labeling of endogenous proteins was not impacted by tagging APEX2 with βarr1. There was no labeling in cells transfected with empty vector and treated with biotin-phenol and H_2_O_2_, nor was there labeling in cells transfected with βarr1-APEX2 but not treated with biotin-phenol and H_2_O_2_ (Figure 3B). Biotinylated proteins were purified via streptavidin agarose pull-down and analyzed by immunoblotting for known βarr1-interacting proteins. As shown in Figure 3B, STAM1, FAK and AIP4, which are known to interact with βarr1 in a CXCL12-dependent manner [14,36], were present. Densitometric analysis from three independent experiments revealed a statistically significant twofold to threefold increase for STAM1 (Figure 3C), FAK (Figure 3D) or AIP4 (Figure 3E) binding at 5 min or 60 min of CXCL12 stimulation relative to the basal condition. STAM1 and AIP4 were also present in APEX2 alone transfected cells, likely by bystander biotinylation; however, they were similar to the levels observed in the basal condition in βarr1-APEX2 transfected cells. FAK biotinylation appeared to be higher in APEX2 alone transfected cells relative to basal and also appeared to increase following CXCL12 stimulation (Figure 3B), however this was not statistically significant when averaged over three independent experiments (Figure 3D). These data are consistent with our results from previous biochemical experiments in which we have shown that CXCL12 stimulation of CXCR4 promotes βarr1 binding to STAM, FAK, or AIP4 [29,30,36]. 

**Figure 4:**
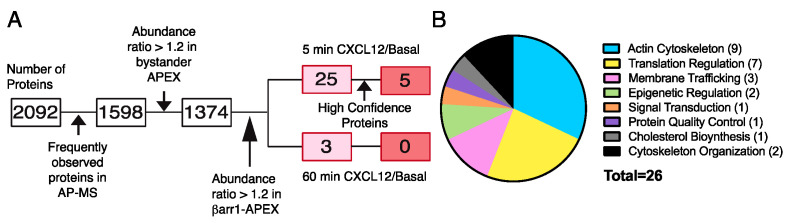
Filtering approach and protein classification of β-arrestin1-interacting proteins. (**A**). Schematic of the filtering approach to select high confidence βarr1-interacting proteins. Number of proteins identified by mass spectrometry totaled approximately 2092 proteins. Proteins frequently observed in affinity purification mass spectrometry (AP-MS) experiments according to the CRAPome database (https://reprint-apms.org/) accessed on 7 January 2022 were removed, reducing the list to ~1598 proteins. Proteins were also removed if they had an abundance ratio >1.2 in any of the bystander APEX conditions, reducing the list to ~1374 proteins. The list of these proteins is provided in File S1, located in the Supplementary Materials. Proteins from the βarr1-APEX transfection condition with an abundance ratio >1.2 at 5 min (25 proteins) or 60 min (3 proteins) with CXCL12 compared with the basal condition were considered potential β-arrestin1-interacting proteins. Abundance ratios from each biological replicate were analyzed by a Student’s paired ratio t-test. Five β-arrestin1-interacting proteins with high confidence (90% confidence interval) were identified. Five proteins, which were identified in one biological replicate (Table 2) were not analyzed further. (**B**). Gene ontology analysis of proteins. Gene ontology classifications were based on the DAVID database (https://david.ncifcrf.gov/tools.jsp) for functional classification or classified based on functional annotation reported in UNIPROT (https://www.uniprot.org/).

Next, frozen aliquots from three independent experiments were processed simultaneously by streptavidin affinity purification followed by tandem mass tag (TMT) mediated quantitative mass spectrometry. We identified approximately ~2100 proteins in each condition. We applied several filtering approaches to remove nonspecific proteins (Figure 4A). We first compared proteins in our list with those commonly reported in the literature as being observed in affinity purification coupled with mass spectrometry experiments [37]. Several proteins (~494) fell into this category (Figure 4A). Furthermore, many of the proteins in the basal condition of either βarr1-APEX2 or APEX2 transfected cells overlapped, consistent with similar bystander labeling. Surprisingly, we did not observe an enrichment of STAM1, FAK, or AIP4, in contrast to the immunoblotting analysis (Figure 3B), although they were present in the mass spectrometry dataset. This is despite the fact that the labeling was identical because we used aliquots from the same samples for the analysis by immunoblotting and mass spectrometry (Figure 3A). One key difference is that the samples processed for immunoblotting were processed immediately after harvesting, whereas the samples processed for mass spectrometry were processed after a short period of storage at −80 °C, which could have compromised the integrity of the samples. Another key difference is that we used different commercial lots of streptavidin-sepharose resin for affinity purification for the immunoblotting versus the mass spectrometry analysis. Lot-to-lot variability of commercial streptavidin-sepharose resin may contribute to significant variability in the quality of data in proximity-dependent biotinylation experiments, even when identical protocols are used [38]. Although we are comparing immunoblotting and mass spectrometry data, this variability could explain the disparate immunoblotting and mass spectrometry results. Other technical reasons are possible that might explain the poor enrichment of these proteins and other proteins in our experimental setup [39].

With this in mind, we considered a protein enriched over basal if the abundance ratio was equal to or greater than 1.2. The data were further filtered by comparing the protein abundance from all samples to the basal condition from cells transfected with βarr1-APEX2. Proteins enriched in any of the APEX2 transfected cells were removed from further analysis. Approximately 300 proteins fell into this category and did not include many known β-arrestin-interacting proteins (Figure 4A). This reduced the candidate list to 25 proteins that were enriched at 5 min and 3 proteins at 60 min of CXCL12 stimulation over the basal condition, with two proteins appearing in both lists (Figure 4A). Of these, 5 proteins were predicted to be βarr1-interacting proteins with high confidence at 5 min (Table 1), none at 60 min. However, 20 proteins were considered to be potential interactors at 5 min and 3 proteins at 60 min, but with low confidence (Table 2). The identified proteins could be placed into the following five main categories according to DAVID gene ontology software: actin cytoskeleton, signal transduction, membrane trafficking, cholesterol biosynthesis, and translation regulation (Figure 4B) [40,41]. These functional categories correlate well with the functional categories previously identified by using traditional mass spectrometry approaches to identify β-arrestin-interacting proteins [16]. However, many of the hits in our screen (Table 1 and Table 2) were not identified in previous screens. This could be reflective of weak or transient interactions that are more amenable to being discovered by proximity labeling when compared with previous methods. 

**Figure 5:**
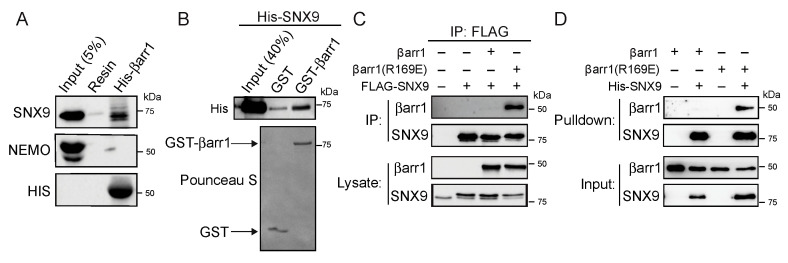
SNX9 interacts directly with β-arrestin1. (**A**) Binding of βarr1 to endogenously expressed SNX9, but not NEMO, in HeLa cells. Immobilized His-βarr1 or empty metal affinity resin was incubated with HeLa cell lysates, and binding reactions were analyzed by immunoblotting. Input represents 5% of the cell lysate used in the binding reaction. NEMO served as a negative control. (**B**) Binding of purified βarr1 to purified SNX9. Equimolar amounts (~1 µM) of GST-βarr1 or GST immobilized on glutathione-sepharose resin were incubated with purified His-SNX9 (0.3 µM), and binding reactions were analyzed by immunoblotting for the His tag. Ponceau S-stained blot shows GST and GST-βarr1 from the binding reaction. Input represents 40% of His-SNX9 used in the binding reaction. (**C**) Preactivated βarr1(R169E) preferentially binds to SNX9. Binding of preactivated βarr1 (R169E) to SNX9 in HEK293 cells transiently transfected with FLAG-SNX9 and wild-type βarr1 or preactivated βarr1-R169E. FLAG immunoprecipitates (IP) and lysates were analyzed by immunoblotting by using antibodies against βarr1 and SNX9. The doublet bands shown in the SNX9 immunoblot represent the FLAG-tagged SNX9 (upper band) and endogenous SNX9 (lower band). (**D**) Immobilized His-SNX9 (0.45 µM) was incubated with wild-type βarr1 or preactivated βarr1-R169E (0.15 µM), and pulldown and input samples were analyzed by immunoblotting by using antibodies against βarr1 and SNX9. Data (**A**–**D**) are representative of three independent experiments.

For follow-up studies, we selected sorting nexin 9 (SNX9), a protein enriched at 5 min of CXCL12 stimulation (Table 1). SNX9 belongs to the sorting nexin family of proteins, which is a family comprised of at least 33 members involved in several aspects of membrane trafficking [42]. SNX9 along with SNX18 and SNX33 are part of a subfamily characterized by a similar domain organization [43]. This family has been linked to plasma membrane remodeling involved in clathrin-dependent and -independent endocytosis [44,45]. To our knowledge, the SNX9-family has not been previously shown to be a target of βarr1. We first examined whether SNX9 interacted with βarr1 in orthogonal biochemical pulldown experiments and by immunoblotting. Bacterial purified His-tagged βarr1 (His-βarr1) immobilized to cobalt resin, precipitated endogenous SNX9 from a cleared cell lysate of HeLa cells when compared with empty resin or nonspecific binding (Figure 5A). His-βarr1 did not precipitate NEMO (Figure 5A), a protein that was observed in the proximity labeling experiment but was removed from further analysis because it was also enriched in APEX transfected cells. To determine whether the interaction between SNX9 and βarr1 is direct, we incubated purified GST-tagged βarr1 (GST-βarr1) with purified His-SNX9 and analyzed binding by immunoblotting. His-SNX9 was robustly precipitated by GST-βarr1 when compared with GST (Figure 5B). These data confirm that βarr1 interacts directly with SNX9 (Figure 5B). 

These biochemical experiments were done in the absence of GPCR stimulation, which is likely required to enhance the affinity of the interaction between βarr1 and SNX9. To address this, we assessed whether the activation of βarr1 regulates the interaction with SNX9. To examine this, we used a preactivated form of βarr1 (βarr1-R169E) [46,47]. Preactivated βarr1 mimics the activated state by disrupting the polar core, which normally keeps arrestins in an inactive state before it binds to ligand-activated and phosphorylated GPCRs [48]. HEK293 cells were transiently transfected with FLAG-tagged SNX9 and wild-type βarr1 or the R169E variant followed by FLAG immunoprecipitation of cleared lysates and immunoblotting. Remarkably, R169E, but not wild-type βarr1, was observed in FLAG immunoprecipitates of cells expressing FLAG-SNX9, indicating that SNX9 prefers to bind to the activated form of βarr1 (Figure 5C). Similarly, purified His-tagged SNX9 preferred preactivated purified βarr1-R169E, not wild-type βarr1 (Figure 5D). These data suggest that SNX9 is a GPCR-regulated β-arr1-interacting partner.

**Figure 6:**
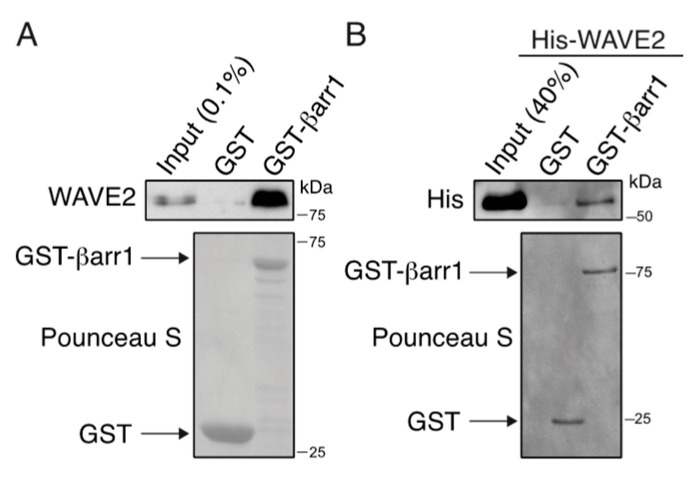
WAVE2 interacts directly with β-arrestin1. (**A**) Binding of βarr1 to endogenously expressed WAVE2 in HeLa cells. Equimolar amounts (~1 µM) of immobilized GST-βarr1 or GST on glutathione-sepharose resin were incubated HeLa cell lysates, and binding reactions were analyzed by immunoblotting. Ponceau S-stained blot shows GST and GST-βarr1 from the binding reaction. Input represents 0.1% of lysate used in the binding reaction. (**B**) Binding of purified WAVE2 to βarr1. Equimolar amounts (~1 µM) of GST-βarr1 or GST immobilized on glutathione-sepharose resin were incubated with HIS-WAVE2 (0.5 µM), and pulldown and input samples were analyzed by immunoblotting. Ponceau S-stained blot shows GST and GST-βarr1 from the binding reaction. Input represents 40% of HIS-WAVE2 used in the binding reaction. Data are representative of 3 independent experiments.

Given the disparate results between our immunoblotting and mass spectrometry results, we decided to follow up on one of the proteins, which, through our filtering approach, was removed from our analysis and, as such, was not defined as a βarr1-interacting partner. For this, we selected WAVE2 (Wiskott–Aldrich syndrome protein (WASP)–homology domain 2; UniProt ID: Q9Y6W5), which showed an enrichment >1.4 at 5 min of CXCL12 stimulation compared with the basal condition, but it was also enriched in the APEX cells alone, or the bystander condition. WAVE2 has not previously been shown to interact with β-arrestin by other mass spectrometric-based discovery approaches [16]. WAVE2 is linked to the regulation of the actin cytoskeleton and cell migration by forming nucleation sites for actin filament formation at the leading edge of cells [49]. In this way, WAVE2 could potentially be relevant to CXCR4 chemotactic signaling at the plasma membrane. Therefore, we selected WAVE2 for follow-up biochemical experiments. Bacterially purified GST-βarr1 or GST alone immobilized to glutathione resin was incubated with a cleared cell lysate of HeLa cells (Figure 6A). Endogenous WAVE2 bound to GST-βarr1, but not GST alone (Figure 6A) and purified His-tagged WAVE2 bound to GST-βarr1, but not GST (Figure 6B). These data confirm that βarr1 interacts with WAVE2 and that the interaction is direct. 

## 3. Discussion

Here, we used APEX2-based proximity labeling coupled with affinity purification and quantitative mass spectrometry to define the GPCR signaling network. We appended APEX2 in-frame to the carboxy-terminus of βarr1 (βarr1-APEX2), in contrast to previous studies that appended APEX2 in-frame to the C-terminus of GPCRs [17,19,26,27]. βarr1-APEX2 interacted with known βarr1 binding partners, and the APEX2 tag did not impact its functional role in GPCR internalization. We showed that βarr1-APEX2 labeled known βarr1-interacting partners following GPCR activation with an agonist as assessed by streptavidin affinity purification and immunoblotting. Parallel samples were analyzed by streptavidin affinity purification, TMT labeling, and quantitative mass spectrometry. We identified several proteins that were increased in abundance with high confidence following CXCL12 stimulation. Biochemical experiments confirmed two novel β-arrestin1-interacting proteins, which have not previously been linked to GPCR or β-arrestin-mediated signaling. Therefore, βarr1-APEX2-based proximity labeling represents a useful unbiased approach to identifying novel players in the GPCR signaling network.

We applied a multilayered filtering approach in identifying GPCR-stimulated βarr1-interacting partners with high confidence (Figure 4). These proteins have not been previously shown to interact with β-arrestins, although, unlike previous studies, we set out to identify proteins that are regulated by CXCR4 stimulation [16]. However, the proteins fall into several functional categories, including the actin cytoskeleton, signal transduction, membrane trafficking, cholesterol and protein biosynthesis (Table 1), consistent with traditional proteomic approaches to identify β-arrestin-interacting proteins [16]. Of these, TAOK1, which is a serine/threonine kinase that mediates activation of the p38 MAP kinase cascade [50], has been previously linked to GPCR signaling [51]. Another protein we identified is SNX9, which is involved in clathrin-dependent and -independent endocytosis through its ability to remodel the plasma membrane and recruit dynamin at late stages of endocytosis [43]. Further, we identified a protein called SYNPO, which is related to synaptopodin, an actin accessory protein found in the brain and kidney podocytes [52]. While localization of these three proteins has been linked to the plasma membrane, where βarr1 is thought to be recruited to CXCR4 (Figure 1), we also identified proteins linked to other cellular functions not necessarily localized to the plasma membrane, such as cholesterol biosynthesis and translation control or protein biosynthesis (Table 1). Our study expands the β-arrestin1 signaling network and opens research avenues for exploring possible GPCR-dependent differences in the β-arrestin interactome.

Although we discovered several proteins with high confidence (Table 1), we selected SNX9 for follow-up biochemical experiments. Similar experiments are required to confirm if the other proteins are genuine βarr1-interacting proteins. Because of the APEX2 labeling radius of 20 nm, it is possible that some of these interactions are indirect, or proximal to βarr1 but not direct interactors. For SNX9, we provide evidence it is a direct interaction because purified His-tagged SNX9 robustly interacted with βarr1 (Figure 5), although we cannot exclude the possibility of an indirect interaction in our experimental setup. Interestingly, the coimmunoprecipitation experiments revealed a strong preference for SNX9 binding to preactivated βarr1 (R169E variant) (Figure 5C,D), suggesting a conformational requirement for the interaction and is consistent with the interaction being regulated by CXCR4 activation and further suggesting that the interaction might be broadly regulated by GPCRs. 

The reason we selected SNX9 for follow-up experiments was in part due to the fact of its known localization to the plasma membrane [45]. Notably, the enrichment of SNX9 was observed at 5 min, suggesting the interaction occurs at or near the plasma membrane where βarr1 mainly localizes following agonist activation of CXCR4 [29]. We have previously shown that βarr1 also colocalizes with CXCR4 on EEA1-positive early endosomes after 30–60 min of CXCL12 stimulation in HeLa cells [14]. SNX9 was not enriched at 60 min of CXCL12 stimulation, suggesting it is not recruited to early endosomes. We used cytosolic APEX as our spatial control to define which compartment binding partners interact with βarr1 with high confidence, adding additional spatial controls to the proximity labeling experiments, such as spatially restricting APEX to the plasma membrane and early endosomes, would be helpful. Further validation experiments are required to precisely determine the subcellular site where SNX9 is recruited following GPCR stimulation. Interestingly, SNX9 has a functional role in membrane trafficking [45], and future experiments will be required to discern the functional outcome of SNX9 on CXCR4 trafficking or signaling.

We identified several enriched proteins that were not determined to be high-confidence interacting proteins (Table 2). Similar to the high-confidence hits, these proteins fall into similar functional categories identified in previous mass spectrometry screens to identify β-arrestin-interacting proteins [16]. Of particular interest is the identification of proteins linked to the actin cytoskeleton, especially given the role of chemokine receptor signaling and β-arrestins in cell migration [29]. One protein, supervillin (SVIL) belongs to the gelsolin family of actin-binding proteins that regulate actin dynamics at the plasma membrane to control cell migration [53]. Interestingly, gelsolin was previously shown to interact with β-arrestins via coimmunoprecipitation and mass spectrometry analysis [16]. Another protein of interest that is linked to cell migration is ARAP1. ARAP1 is a phospholipid-regulated GAP for Arf and especially Rho small GTPases that control the actin cytoskeleton during cell migration [54]. ARAP1 was the only protein we identified to have increased abundance at 5 min and 60 min CXCL12 stimulation (Table 2). We have yet to verify whether any of these proteins interact with βarr1.

We focused our analysis on identifying βarr1-interacting proteins following agonist activation of CXCR4. Surprisingly, this resulted in a small number of high-confidence or low-confidence hits (Table 1 and Table 2). However, we identified many proteins by mass spectrometry that were not enriched relative to the basal βarr1-APEX2 condition (Figure 4). Many of these proteins were identified in previous traditional mass spectrometry screens to identify β-arrestin-interacting proteins [16]. Although we validated our labeling and overall approach (Figure 1) by immunoblotting of known βarr1-interacting proteins following GPCR stimulation (Figure 3B) this was not matched when interacting proteins were detected by mass spectrometry (Figure 4). Several technical reasons might explain the poor enrichment of these proteins and other proteins in our experimental setup related to the mass spectrometry approach [38,39]. Our dataset also includes proteins such as WAVE2, a false negative, which was removed from further analysis because it was also enriched in the APEX2-transfected cells (Figure 4). We decided to pursue WAVE2 for validation experiments because of its known localization at the plasma membrane and its functional connection to cell migration. However, although we cross-validated that it interacts with βarr1 by pulldown assays (Figure 6), we have yet to validate its functional role in CXCR4 signaling. Nevertheless, this example underscores the possibility that our dataset contains additional βarr1-interacting proteins. 

We selected HeLa cells for the proximity labeling because they express high levels of endogenous CXCR4, therefore not requiring the exogenous expression of this GPCR, which could confound the interpretation of our data due to overexpression artifacts. However, HeLa cells also express the scavenger receptor ACKR3 (a.k.a. CXCR7), which also binds to CXCL12, the cognate ligand for CXCR4 [55]. ACKR3 is mostly a nonsignaling receptor as it does not couple with G proteins [56]. However, ACKR3 is believed to couple with β-arrestins; therefore we cannot exclude the possibility that the interacting partners described here are due in part to βarr1-interacting with CXCL12-stimulated ACKR3 [57]. A potential caveat of our study is that we transiently overexpressed βarr1-APEX2, which could potentially identify proteins not seen in an endogenous setting. Stable expression of βarr1-APEX2 in β-arrestin1/2 deficient HEK293 [32,33] or HeLa [58] cells would be closer to resembling an endogenous setting and more precisely identify interacting partners. We propose that β-arrestin-APEX2 proximity labeling is useful to detect differential enrichment of proteins following the activation of different GPCRs and/or different ligands at the same GPCR. 

In summary, we demonstrate that proximity labeling by appending APEX2 to βarr1 is useful to discover novel β-arrestin-interacting proteins following GPCR activation. Because GPCRs can signal via G protein- and β-arrestin-dependent pathways, this approach may also be useful to selectively define β-arrestin-dependent signaling by functionally selective ligands. It is expected this approach will reveal new functional insights into β-arrestin-dependent signaling by GPCRs.

## 4. Materials and Methods

### 4.1. Cell Culture, Antibodies, and Reagents

HEK293 cells were from Microbix (Toronto, ON, Canada). HeLa and COS-1 cells were from American Type Culture Collection (Manassas, VA, USA). The β-arrestin-1 and -2-deficient HEK293 cells were kindly provided by Asuka Inoue (Tohoku University, Sendai, Japan) [32,33]. Cells were maintained in DMEM (cat. no. D5796; Sigma, St. Louis, MO, USA) supplemented with 10% FBS (cat. no. FB-02; Omega Scientific, Riverside, MO, USA). The rabbit anti-STAM1 (cat. no. 12434-1-AP), anti-FAK (cat. no. 12636-1-AP), anti-SNX9 (cat. 15721-1-AP), and anti-NEMO (cat. no. 18474-1-AP) antibodies were from Proteintech (Rosemont, IL, USA). The rabbit anti-β-arrestin1 (cat. no. 12697) and anti-WAVE2 (cat. no. 3659) antibodies were from Cell Signaling Technologies (Danvers, MA, USA). The mouse anti-GAPDH antibody (cat. no. ab9482) and anti-AIP4 antibody (cat. no. ab108515) were from Abcam (Cambridge, UK). Streptavidin-HRP (cat. no. 18-152), the mouse anti-FLAG M2 antibody (cat. no. F4049), mouse anti-FLAG M2 alkaline phosphatase antibody (cat. no. A9469), biotinyl tyramide (cat. no. SML2135), hydrogen peroxide solution 30% (*w*/*w*) (cat. no. H1009), sodium ascorbate (cat. no. A4034), Trolox (cat. no. 238813), and sodium azide (cat. no. S2002) were from Sigma-Aldrich (St. Louis, MO, USA). CXCL12 was from Protein Foundry (Milwaukee, WI, USA). Streptavidin agarose resin (cat. no. 20353) was from Thermo Scientific (Rockford, IL, USA). TALON^®^ metal affinity resin (cat. no. 635501) and glutathione-superflow resin (cat. no. 635607) were from Takara Bio USA. 

### 4.2. DNA Plasmids 

The plasmids encoding GST-βarr1, βarr1-wildtype, or R169E variant were previously described [14]. The βarr1-APEX2 plasmid was made by using NEBuilder HiFi DNA assembly (New England BioLabs, Ipswich, MA, USA; cat. no. E5520) of a pcDNA backbone fragment containing the βarr1 coding region amplified by PCR from βarrestin1-FLAG plasmid [29] and an APEX2 fragment amplified by PCR from pcDNA3-APEX2-NES plasmid, which was a gift from Alice Ting (Addgene plasmid #49386). The His-SNX9-pET15b plasmid was a gift from Sandra Schmid (Addgene plasmid #34690). The His-βarr1 plasmid was made by using NEBuilder HiFi DNA assembly of the pET15b backbone fragment and a fragment of the βarr1 coding region amplified by PCR from pcDNA3 βarr1 [30]. The His-WAVE2 plasmid was made by using NEBuilder HiFi DNA assembly of the pET15b backbone and a WAVE2 fragment amplified by PCR from pCellFree_03 WAVE2, which was a gift from Kirill Alexandrov (Addgene plasmid #67098). The FLAG-SNX9 plasmid was made by using NEBuilder HiFi DNA assembly of a fragment of the pCMV10 backbone and an SNX9 fragment amplified by PCR from His-SNX9-pET15b. The sequence of the primers used for generating new plasmids described in this study are listed in File S2. All plasmids were confirmed by dideoxy sequencing.

### 4.3. Expression and Purification of Recombinant Proteins

Untagged wild-type βarr1 or βarr1-R169E variant and His-tagged SNX9, WAVE2, or βarr1 were expressed in *Escherichia coli* (*E. coli*) BL-21 (DE3) cells and purified by using protocols we have previously described [59,60,61]. Briefly, for untagged βarr1, the cultures were induced at OD_600_ ~0.4–0.6 with 250 µM isopropyl 1-thio-β-D-galactopyranoside (IPTG) for 4 h at 30 °C. For His-tagged SNX9, WAVE2, or βarr1, the cultures were induced at OD_600_ ~0.4–0.6 with 1 mM IPTG for 2–3 h at 30 °C for WAVE2 and SNX9 or 18 °C for βarr1. Untagged βarr1 and βarr1-R169E were pelleted and resuspended in lysis buffer 1 (50 mM Tris-HCl, pH 8.0, 5 mM EGTA, 2 mM benzamidine, 2 mM DTT and 10 µg/mL leupeptin, 10 µg/mL pepstatin A, and 10 µg/mL aprotinin) followed by ammonium sulfate precipitation. The protein pellet was dissolved in column buffer (10 mM Tris, 2 mM EDTA, 2 mM EGTA, pH 7.5), which was immediately followed by sequential chromatography on heparin-sepharose (GE Life Sciences, Chicago, IL, USA, cat. no. 17-0407-01) and Q-sepharose (GE Life Sciences, cat. no. 17-1154-01) columns. For His-SNX9, His-WAVE2, and His-βarr1 bacterial cell pellets were resuspended in lysis buffer 2 (20 mM Tris-HCl, pH 7.4, 150 mM NaCl, 0.1% Triton X-100, 1 mM DDT, 10 μg/mL leupeptin, 10 μg/mL pepstatin A, and 10 μg/mL aprotinin), and incubated with metal affinity resin overnight while gently rocking at 4 °C. The next day, the resin was packed into an empty column and washed with increasing concentrations of imidazole (5 mM–500 mM) in Tris-buffered saline (TBS; 20 mM Tris-HCl, pH 7.5, and 150 mM NaCl). Fractions were analyzed by 10% SDS-PAGE and Coomassie blue staining. Proteins were concentrated by using Amicon Ultra-0.5 centrifugal concentrators with a molecular weight cutoff of 10,000 Dalton (cat. no. UFC500396) [14]. GST and GST-β-arrestin1 were purified exactly as previously described [62].

### 4.4. Pulldown Assay

His-tagged proteins immobilized to metal affinity resin or GST-tagged proteins immobilized to glutathione resin were used in pulldown binding assays with cleared cell lysates or purified proteins by using protocols we have previously described [14]. Immobilized His-βarr1 was incubated with cleared cell lysates prepared from HeLa cells (400–600 µg) and His-SNX9 was incubated with equimolar amounts (1 µM) of purified wild-type βarr1 or βarr1-R169E for 2 h at 4 °C. GST or GST-βarr1 (1 µM) was incubated with HeLa cell lysates (400–600 µg), purified His-SNX9 (0.3 µM) or His-WAVE2 (0.5 µM) for ~18 h at 4 °C. Samples were washed three times in ice-cold binding buffer (20 mM Tris-HCl, pH 7.5, 150 mM NaCl, 0.1% Triton X-100, 10 μg/mL leupeptin, 10 μg/mL pepstatin A, and 10 μg/mL aprotinin) and bound proteins were eluted in 2× sample buffer (8% SDS, 10% glycerol, 5% β-mercaptoethanol, 37.5 mm Tris-HCl, pH 6.5, 0.003% (*w*/*v*) bromophenol blue) for GST-tagged proteins or 200 mM imidazole for His-tagged proteins. Samples were analyzed by SDS-PAGE and immunoblotting. 

### 4.5. Coimmunoprecipitation 

COS-1 cells grown in 10-cm plates were transiently transfected with FLAG-STAM1 or empty vector (pCMV10) and βarr1-APEX2, βarr1-GFP or empty vector (pcDNA3) using polyethyleneimine (PEI) (Polysciences Inc., Warrington, PA, USA, cat No. 23966-1). HEK293 cells were transiently transfected with FLAG-SNX9 and wild-type βarr1, βarr1 variant R169E, or empty vector (pcDNA3) using PEI. Twenty-four hours posttransfection, COS-1 cells were lysed in immunoprecipitation (IP) buffer (50 mM Tris-HCl, pH 7.5, 150 mM NaCl, 0.5% NP-40, and 10 μg/mL leupeptin, 10 μg/mL pepstatin A, and 10 μg/mL aprotinin), and HEK293 cells were lysed in lysis buffer 2, described above. Cellular debris was cleared by centrifugation at 21,000× *g* for 30 min at 4 °C. The protein concentration of supernatants was determined by using the 660-nm protein assay kit from Pierce (cat. no. 22660), according to the manufacturer’s instructions. Equal amounts of lysates were incubated with the M2 anti-FLAG tag antibody overnight at 4 °C while gently rocking it. Twenty μL of a 50% slurry of protein G agarose resin (Sigma-Aldrich; cat. no. 11243233001) that was extensively equilibrated, respectively, in ice-cold IP or lysis buffer 2 was added to each sample for 1 h at 4 °C. Samples were washed three times with ice-cold IP buffer or lysis buffer 2, respectively, and bound proteins were eluted in 20 μL of 2× sample buffer (8% SDS, 10% glycerol, 5% β-mercaptoethanol, 37.5 mM Tris-HCl, pH 6.5, 0.003% bromophenol blue) or with 200 µg/mL FLAG peptide (Sigma-Aldrich; cat. no. F3290). Samples were analyzed by SDS-PAGE and immunoblotting.

### 4.6. Biotin Labeling

HeLa cells grown on 10-cm dishes were transiently transfected with β-arrestin1-APEX2, APEX2, or empty vector (pcDNA3) by using PEI. Twenty-four h later, cells were serum-starved for 3 h at 37 °C with DMEM containing 20 mM HEPES. This medium was replaced with 10 mL of the same medium containing 500 µM biotin phenol followed by incubation at 37 °C for 1 h. Cells were then left untreated or treated with 50-nM CXCL12 for 5 min or 60 min at 37 °C. Biotin labeling was initiated by adding 10 mL of DMEM supplemented with 20-mM HEPES containing 2 mM H_2_O_2_ to each dish followed by brief agitation to achieve a final concentration of 1 mM H_2_O_2_. Reactions were maintained at room temperature for 1 min. One dish of cells transfected with β-arrestin1-APEX2 was excluded from biotin phenol incubation and H_2_O_2_ treatment and was set as a negative control. To quench the reaction, the labeling medium was aspirated, and cells were washed three times with ice-cold, freshly made quenching solution (10 mM sodium ascorbate, 5 mM Trolox, and 10 mM sodium azide in DPBS). Cells were then incubated in 10 mL of quenching solution for 20 min on ice. Quenching solution was aspirated and cells were lysed in 500 µL ice-cold lysis buffer 3 [50 mM Tris-HCl, pH 7.5, 150 mM, NaCl, 0.1% (wt/vol) SDS, 0.5% (wt/vol) sodium deoxycholate and 1% (vol/vol) Triton X-100 supplemented with 1× ProBlock Gold protease inhibitor cocktail (GoldBio, St. Louis, MO, USA, cat. no. GB-108-5), 1 mM PMSF, 1 mM sodium azide, 10 mM sodium ascorbate, 1 mM DTT, and 1 mM Trolox]. Lysates were transferred to prechilled microcentrifuge tubes, briefly sonicated and clarified by centrifugation at 14,000× *g* for 20 min at 4 °C. The protein concentration of supernatants was determined by using the 660-nm protein assay kit from Pierce, according to the manufacturer’s instructions. One aliquot of the supernatants (250 µg) was immediately subject to streptavidin affinity purification. A second aliquot was flash-frozen and stored at −80 °C. 

### 4.7. Streptavidin Affinity Purification of Biotinylated Proteins

Equal amounts of supernatants (250 µg in 500 µL) were incubated with 75 µL of a 50:50 slurry of streptavidin agarose resin under gentle agitation at 4 °C overnight. Samples were washed twice with 1 mL of ice-cold buffer 3, once with 1 mL of 2 M urea in 10 mM Tris-HCl pH 8.0, and twice again with 1 mL of buffer 3. Biotinylated proteins were eluted by incubating the resin with 30 µL of 2× sample buffer supplemented with 20 mM DTT and 2 mM biotin and heated at 100 °C for 5 min. Samples were analyzed by SDS-PAGE and immunoblot or streptavidin-HRP analysis. 

### 4.8. Sample Preparation for Mass Spectrometry Analysis

Frozen aliquots of supernatants from three independent experiments were processed simultaneously for streptavidin affinity purification and mass spectrometry analysis. Samples were thawed on ice and equal amounts of supernatants (250 µg in 500 µL) were incubated with 75 µL of a 50:50 slurry of streptavidin agarose resin under gentle agitation at 4 °C overnight. Samples were washed three times with 1 mL of ice-cold buffer 3, and three times with 1 mL of ice-cold 100 mM sodium phosphate, pH 8 containing 4 M urea. Samples were immediately processed for mass spectrometry analysis as described in detail in the Supplementary Methods.

### 4.9. Receptor Surface Expression

Receptor internalization was examined by whole-cell ELISA assay, as previously described [14,31]. Briefly, β-arrestin-deficient HEK293 cells grown on 10-cm dishes transiently transfected with FLAG-β_2_AR and βarr1-APEX2 or pcDNA were seeded in DMEM containing 10% FBS onto 24-well plates coated with poly-L-lysine (Sigma; cat. no. P1399). The next day, the medium from each well was replaced with DMEM supplemented with 20 mM HEPES, and cells were stimulated with either vehicle or 10 µM isoproterenol for 30 min. Cells were rinsed once with ice-cold TBS (20 mM Tris-HCl, pH 7.5, 150 mM NaCl) and immediately fixed with 3.7% formaldehyde (Sigma; catalog no.: F8775) for 5 min. After three washes with TBS, cells were incubated for 45 min at room temperature in TBS containing 1% bovine serum albumin (TBS-BSA; GoldBio, cat. no. A42010) and then incubated with an anti-FLAG alkaline phosphatase–conjugated antibody at a dilution of 1:1000 in TBS-BSA for 1 h at room temperature. Cells were washed three times with TBS followed by incubation with a developing solution until the appearance of a light yellow color. Reactions were quenched by transferring 100 μL of this solution to a 96-well plate containing 100 μL of 0.4 M NaOH. The 96-well plate was read at 405 nm single end-point reading. Receptor internalization was calculated by subtracting the absorbance from cells treated with isoproterenol divided by the absorbance from cells treated with the vehicle from one. Data represent mean ± the standard deviation from three independent experiments performed in triplicate.

## Figures and Tables

**Table 1:** High confidence proximity labeled proteins. High-confidence proteins (90% confidence interval) are classified based on their functional annotation. The abundance ratios are the average from three independent experiments ± the standard deviation.

Functional Annotation	Symbol	Name	Abundance Ratio (CXCL12/Basal)	UniProt Accession Number
Actin Cytoskeleton	SYNPO	SYNPO protein	1.33 ± 0.42	A7MD96
Signal Transduction	TAOK1	Serine/threonine-protein kinase TAO1 (TAO—thousand and one amino acid)	1.24 ± 0.24	Q7L7X3
Membrane Trafficking	SNX9	Sorting nexin 9	1.23 ± 0.19	Q9Y5X1
Cholesterol Biosynthesis	DHCR24	Delta(24)-sterol reductase	1.24 ± 0.08	Q15392
Translation Regulation	BZW2	eIF5-mimic protein 1	1.22 ± 0.17	Q9Y6E2

**Table 2:** Proximity labeled proteins that were not considered to be high confidence β-arrestin1-interacting proteins. Proteins are classified based on their functional annotation. The abundance ratios are the average from three independent experiments ± the standard deviation. One asterisk (*) indicates proteins identified at 60 min of CXCL12 stimulation and in one biological replicate. Two asterisks (**) indicates proteins identified in one biological replicate. Three asterisks (***) indicates proteins identified at 5 min and 60 min of CXCL12 stimulation.

Functional Annotation	Symbol	Name	Abundance Ratio (CXCL12/Basal)	UniProt Accession Number	
Actin Cytoskeleton and Cell Motility	SVIL	Supervillin	1.23 ± 0.23	O95425	
	ARAP1	Arf-GAP with Rho-GAP domain, ANK repeat and PH domain-containing protein 1	1.24 ± 0.08	Q96P48	***
	SPAG1	Sperm associated antigen 1, isoform CRA_b	1.42 ± 0.40	A0A024R9D8	
	DSC1	Desmocollin-1	1.42 ± 0.68	Q08554	
	NEXN	Nexilin	1.30	Q0ZGT2	**
	FMNL1	Formin-like protein 1	1.24 ± 0.40	O95466	
	CD44	CD44 antigen	1.22 ± 0.30	P16070	
	CAP2	Adenylyl cyclase-associated protein 2	1.23	P40123	**
Membrane Trafficking	VAC14	Protein VAC14 homolog	1.22	Q08AM6	***
	HPCAL1	Hippocalcin-like protein 1	1.26	P37235	*
Protein Quality Control	AGAP3	Arf-GAP with GTPase, ANK repeat and PH domain-containing protein 3	1.21 ± 0.15	Q96P47	
Translation Regulation	SF4	Splicing factor 4	1.26 ± 0.01	Q08170	
	CUX1	Homeobox protein cut-like 1	1.26 ± 0.11	P39880	
	LCMT2	cDNA FLJ76457, highly similar to Homo sapiens leucine carboxyl methyltransferase 2 (LCMT2), mRNA	1.31 ± 0.11	A8K972/O60294	
	ZNF622	Zinc finger protein 622	1.32 ± 0.58	Q969S3	
	WDR33	pre-mRNA 3′ end processing protein WDR33	1.21	Q9C0J8	**
	RPL37	60S ribosomal protein L37	1.36	P61927	**
Epigenetic Regulation	PPHLN1	Periphilin-1	1.27 ± 0.01	Q8NEY8/F8W0Q9	
	ANP32E	Acidic leucine-rich nuclear phosphoprotein 32 family member E	3.97 ± 4.26	Q9BTT0	
Cytoskeleton Organization	CSKI2	Caskin-2	1.25 ± 0.35	Q8WXE0	
	FLJ61294	cDNA FLJ61294, highly similar to Keratin, type I cytoskeletal 17	1.23 ± 0.19	B4DJM5	

## Data Availability

The raw mass spectrometry data presented in this study are available by request from the corresponding author (A.M.).

## Supplementary Materials

The supporting information can be downloaded at https://www.mdpi.com/article/10.3390/ijms24043285/s1.
