# Supplementary material for: Proximity Labeling to Identify β-Arrestin1 Binding Partners Downstream of Ligand-Activated G Protein-Coupled Receptors
Source: Int J Mol Sci. 2023 Feb 7;24(4):3285. doi: 10.3390/ijms24043285 (PMC9967311; doi:10.3390/ijms24043285)
Supplement: Supplementary file 1 [file ijms-24-03285-s001.zip › supplementary methods - Tables S1-S4.pdf]

Supplemental Methods:

Supporting Information:

Proximity labeling to identify β-arrestin1 binding partners downstream of ligand-activated G protein-coupled receptors

Ya Zhuo, Valeria Robleto and Adriano Marchese

SUPPLEMENTAL METHODS

Proximity labeled proteins analyzed by mass spectrometry

Sample Digestion and Desalting

24 samples containing streptavidin beads, washed with 100 mM phosphate buffer with 4M urea, were submitted for proteolytic digestion directly on the beads. Liquid was decanted from the beads and the beads were then resuspended in 50 µL fresh 2 M urea, 50 mM ammonium bicarbonate, 8% acetonitrile (ACN) with 1 µL MS grade LysC stock (2 mg/mL, Thermo). Tubes were vortexed and then incubated with shaking at 37 °C and 1400 rpm for 3 hours. Samples were then diluted 1:4 with 50 mM ammonium bicarbonate, followed by the addition of 1µg MS grade trypsin per tube (Thermo). Digestion proceeded at 37 °C and 1400 rpm overnight. 10 µL 10% trifluoroacetic acid (TFA) was added to each tube to a final concentration of 0.5% TFA to quench digestion. Peptides were then desalted and cleaned using SOLAµ Solid-Phase Extraction (Thermo) according to the manufacturer's instructions and then dried under vacuum.

TMT10-plex Labeling and Cleanup

Each sample was resuspended in 120 µL 50 mM triethylammonium bicarbonate (TEAB). 10 µL of each sample was then assayed via the Pierce Peptide Fluorometric Quantitation Assay (Thermo) according to the manufacturer's instructions. Sample peptide concentrations ranged from 9.1 to 17.0 ng/µL, excluding the negative controls. 8 tubes of 0.8 mg Tandem Mass Tag 10-plex (Thermo) isobaric label stocks were each dissolved in 41 µL 100% acetonitrile (ACN) per biological replicate, all TMT10-plex reagents were indicated as belonging to the same lot and quantitation value correction datasheet. The 24 peptide samples were labeled with the full volume of TMT10-plex reagents at 25 °C for 1 hour according to Table S1.

Table S1. Experimental Design for TMT labels

Condition	pcDNA3	β-Arrestin 1	APEX2	Biotin phenol, H_2O_2 reaction Time (min)	TMT10-plex Tag
			**Bio Rep 1**		
**1.1**	+	+	+	0	126
**1.2**	+	+	+	5	127N
**1.3**	+	+	+	60	127C
**1.4**	+	-	+	0	128N
**1.5**	+	-	+	5	128C
**1.6**	+	-	+	60	129N
**1.7**	+	+	+	-	130N
**1.8**	+	-	-	-	131
			**Bio Rep 2**		
**2.1**	+	+	+	0	126
**2.2**	+	+	+	5	127N
**2.3**	+	+	+	60	127C
**2.4**	+	-	+	0	128N

Supplemental Methods:

**2.5**	+	-	+	5	128C
**2.6**	+	-	+	60	129N
**2.7**	+	+	+	-	130N
**2.8**	+	-	-	-	131
**Bio Rep 3**					
**3.1**	+	+	+	0	126
**3.2**	+	+	+	5	127N
**3.3**	+	+	+	60	127C
**3.4**	+	-	+	0	128N
**3.5**	+	-	+	5	128C
**3.6**	+	-	+	60	129N
**3.7**	+	+	+	-	130N
**3.8**	+	-	-	-	131

The TMT labeling reaction was quenched with the addition of 8 µL 5% hydroxylamine and mixing at 25°C for 15 minutes. Labeled peptide samples were then combined into new tubes per biological replicate according to Table 1. 150 µL of each of the three pooled samples was aliquoted and set aside. The remaining volume of the 3 pooled aliquots were then dried and each resuspended in 300 µL 0.1% TFA. Samples were then loaded onto pre-conditioned high-pH reverse-phase columns (Thermo), washed with 0.1% triethylamine (TEA) in 5% ACN. Peptides were then fractionated with 300 µL solvent per fraction with increasing acetonitrile content according to Table S2.

**Table S2. Experimental Design for high-pH fractionation**			
Fraction #	% ACN	Fraction #	% ACN
1	10%	7	30%
2	12.5%	8	35%
3	15%	9	40%
4	17.5%	10	50%
5	20%	11	65%
6	25%	12	80%

Fractions were then paired and combined following each row in Table 2 (*i.e.* Fractions 1 and 7 were combined, fractions 2 and 8 were combined, etc.). All combined fraction samples and the unfractionated aliquots were dried under vacuum. Unfractionated peptides were stored at -80 °C. Combined fraction samples were reconstituted in 65 µL 2% ACN, 0.1% formic acid for LC-MS analysis.

Mass Spectrometry Analysis

Each TMT set was analyzed on a Thermo Scientific Orbitrap Fusion Lumos MS via 3 technical replicate injections using a data-dependent acquisition (DDA), multi-notch isolation instrument method outlined in Table S3. The 18 combined fraction samples were queued for analysis in randomized order within a technical replicate block. 250 ng of HeLa cell lysate were inserted before each of the technical replicate blocks, with one injection following the final sample injection. MS data were analyzed using Proteome Discoverer 2.2 (Thermo) platform as outlined in the Table S4. Protein identifications were filtered to include only those proteins identified by two or more unique peptides identified and ranked as high confidence.

**Table S3.** Chromatography and MS instrument acquisition settings for TMT10-plex MS			
Sample Volume	5 µL	***Isolation Window***	0.7 m/z
Stationary Phase	C_{18}	***MS^2 AGC Target***	1e4
LC Solvent A	100% H2O,	***MS^2 Maximum IT***	50 ms

Supplemental Methods:

	0.1% formic acid		
LC Solvent B	80% ACN,<br>0.1% formic acid	***Normalized Collision Energy***	35
Gradient Ramp<br>***Duration***<br>***Flow Rate***	6-36% B<br>71 minutes<br>300 nL/min	***Minimum Intensity Req.***	5000
Mass Spectrometer	Thermo Orbitrap Fusion Lumos	***Dynamic Exclusion***	60.0 s
Spray Voltage	2.1 kV	***MS^3 acquisition***	Multi-Notch Isolation
In-Source CID	0.0 eV	***MS^3 Fragmentation***	HCD
MS^1 scan range	375-1500 m/z	***MS^3 Detection***	Orbitrap
MS^1 resolution	120,000 @ 400 *m/z*	***MS^3 scan range***	100-500 *m/z*
MS^1 AGC Target	4e5	***MS^3 resolution***	50,000 @ 400 *m/z*
MS^1 Maximum IT	50 ms	***MS^3 Isolation Window***	2.0 m/z
MS^2 acquisition	Data dependent, 3 s cycle time, Centroid	***MS^3 AGC Target***	1e5
MS^2 Fragmentation	CID	***MS^3 Maximum IT***	105 ms
MS^2 Detection	Ion Trap	***Normalized Collision Energy***	65
MS^2 fixed first mass	120 *m/z*	***Stepped Collision Energy***	5

Table S4. DDA search parameters and TMT Reporter Ions Quantitation (Orbitrap Fusion Lumos)

Platform	ProteomeDiscoverer 2.2	***Reporter Quantitation***	20 ppm integration tolerance
Search Algorithms	SequestHT	***Abundance Measurement***	Automatic (S/N)
Validation	Percolator<br>Peptide Validator<br>Protein FDR Validator	***Normalization***	On β-arrestin 1 (P49407) Signal
Database	UniProt; Human; created 10/03/2017	***Scaling***	No Scaling
Digest	Trypsin (full)<br>2 Missed Cleavages Allowed	***Co-Isolation Threshold***	30
Precursor mass tolerance	10 ppm	***Target FDR (Strict) for PSMs:***	0.01
Fragment mass tolerance	0.6 Da	***Target FDR (Relaxed) for PSMs:***	0.05
Static Modifications	Carbamidomethyl (C), TMT10plex (N-terminus)	***Target FDR (Strict) for Peptides:***	0.01
Dynamic Modifications	Oxidation (M), Acetylation (N-terminus), TMT10plex (K)	***Target FDR (Relaxed) for Peptides:***	0.05
